# Mcl-1 is an important therapeutic target for oral squamous cell carcinomas

**DOI:** 10.18632/oncotarget.3932

**Published:** 2015-05-14

**Authors:** Santanu Maji, Sabindra K Samal, Laxmipriya Pattanaik, Swagatika Panda, Bridget A. Quinn, Swadesh K. Das, Devanand Sarkar, Maurizio Pellecchia, Paul B. Fisher, Rupesh Dash

**Affiliations:** ^1^ Institute of Life Sciences, Bhubaneswar, Odisha, India; ^2^ Manipal University, Karnataka, India; ^3^ Department of Oral Pathology & Microbiology, Institute of Dental Sciences, ‘Siksha O Anusandhan’ University, Bhubaneswar, Odisha, India; ^4^ Department of Human and Molecular Genetics, Virginia Commonwealth University, School of Medicine, Richmond, Virginia, USA; ^5^ VCU Institute of Molecular Medicine, Virginia Commonwealth University, School of Medicine, Richmond, Virginia, USA; ^6^ VCU Massey Cancer Center, Virginia Commonwealth University, School of Medicine, Richmond, Virginia, USA; ^7^ Sanford-Burnham Medical Research Institute, La Jolla, California, USA

**Keywords:** Mcl-1, OSCC, mitophagy, sabutoclax, 4-NQO

## Abstract

Oral and oropharyngeal cancers are the sixth most common cancers worldwide. Despite intensive investigation, oral squamous cell carcinomas (OSCC) represent a clinical challenge resulting in significant morbidity and mortality. Resistance to cell death is common in OSCC and is often mediated by the Bcl-2 family proteins. Among all anti-apoptotic Bcl-2 family members, Mcl-1 functions as a major survival factor, particularly in solid cancers. Despite the confirmed importance of Mcl-1 in several neoplasms, the role of Mcl-1 in OSCC survival has yet to be explored. In this study, we found that knocking down Mcl-1 sensitized OSCC cells to ABT-737, which binds to Bcl-2/Bcl-x_L_ but not Mcl-1. We report for the first time that a BH3 mimetic, Sabutoclax, which functions as an inhibitor of all anti-apoptotic Bcl-2 proteins, induced cancer-specific cell death in an Mcl-1-dependent manner through both apoptosis and toxic mitophagy. *In vivo* studies demonstrated that Sabutoclax alone decreased tumor growth in a carcinogen-induced tongue OSCC mouse model. In a combination regimen, Sabutoclax and COX-2 inhibitor, Celecoxib, synergistically inhibited the growth of OSCC *in vitro* and also significantly reduced OSCC tumor growth *in vivo*. Overall, these results identify Mcl-1 as a therapeutic prospective target in OSCC.

## INTRODUCTION

Oral cancer and oropharyngeal cancers are now considered common cancers with an estimated 2.23 million deaths in 2008 [1]. Annually, 40, 000 new cases of oral cancer are reported in the United States whereas in India the incidence is approximately 80, 000 per year. More than 90% of all oral cancers are Oral Squamous Cell Carcinomas (OSCC) [2]. Poor habits like smoking and consumption of smokeless tobacco (gutkha and betel nut) are the major risk factors for OSCC. Oral carcinogenesis occurs through two major pathways: I) transformation of normal epithelium to dysplastic lesions and II) progression of these dysplastic lesions to invasive squamous carcinomas. These transformation events are mediated by the dysfunction of multiple genes, including the Bcl-2 family members and Cyclooxygenases [3].

Bcl-2 family proteins play a critical role in the progression of various neoplasms. The Bcl-2 family can be broadly divided into anti-apoptotic and pro-apoptotic members. The anti-apoptotic proteins (Bcl-2, Bcl-x_L_, Mcl-1, Bcl-w and A1) contain BH1 to BH4 domains. The pro-apoptotic members are further divided into two subgroups. One group (Bax and Bak) contains BH1 to BH3 domains and the other group (NOXA, PUMA, Bim and Bid) contains only the BH3 domain [4]. Among the anti-apoptotic Bcl-2 family members, Mcl-1 plays a major role in tumorigenesis and chemoresistance, particularly in solid cancers [5, 6], distinguishing it as a potentially important therapeutic target. In spite of significant efforts to develop inhibitors of the anti-apoptotic Bcl-2 proteins, many of those developed to date only effectively inhibit Bcl-2 and Bcl-x_L_, but not Mcl-1. Most such inhibitors fall into the class of compounds known as BH3 mimetics, which are small molecules that mimic the BH3 domain of pro-apoptotic Bcl-2 proteins. One currently used BH3 mimetic, ABT-737 and its clinical counterpart ABT-263, display-limited affinity towards Mcl-1 [7]. This could be attributed to the unique structure of Mcl-1 as compared to Bcl-2 or Bcl-x_L_ [8]. Using NMR binding assays and computational studies, an Apogossypol derivative, Sabutoclax, was identified and found to bind to Bcl-x_L_, Bcl-2, and Mcl-1 with IC_50_ values of 190, 360, and 520 nmol/L, respectively [9]. We have previously shown that the Mcl-1 antagonist Sabutoclax alone or in combination with the tumor suppressor *mda*-7/IL-24 induces cancer-specific cell death in human prostate carcinomas (PC) [9, 10]. Furthermore, Sabutoclax alone inhibited both primary tumor growth as well as the metastatic spread of castration-resistant PC in transgenic mice that spontaneously develop prostate adenocarcinomas [11]. Interestingly, it was also found that Sabutoclax enhanced sensitivity to tyrosine kinase inhibitors in leukemia stem cells, whose survival is mostly dependent upon expression of the Bcl-2 family of anti-apoptotic proteins [12].

Earlier studies evaluating the clinicopathological association of Mcl-1 with OSCC showed that Mcl-1 overexpression in OSCC is predominantly associated with a less favorable outcome [13]. Similarly, it was found that the HDAC inhibitor vorinostat sensitizes HNSCC cells to ABT-737-induced cell death by down regulating Mcl-1 [14]. Based on these reports, we hypothesized that Mcl-1 could be a major survival factor in OSCC. In this study, we report for the first time that pharmacological inhibition of Mcl-1 using Sabutoclax induced cancer-specific cell death in OSCC both *in vitro* and *in vivo*. Cell death was attributed to Bak-mediated apoptosis and Bnip3-(BCL2/adenovirus E1B 19 kDa protein-interacting protein 3) induced mitophagy. As combination therapy is extremely important for the successful treatment of cancer, we explored additional agents of potential benefit to combine with Sabutoclax in OSCC. Previous literature in OSCC clearly showed an important role for COX-2 in OSCC tumor development and progression. For this reason, we evaluated Sabutoclax in combination with a COX-2 inhibitor, Celecoxib, and found that this novel combination synergistically inhibited the growth of OSCC both *in vitro* and *in vivo*. These studies reinforce the importance of Mcl-1 in OSCC and provide preclinical data in support of Sabutoclax and Celecoxib as a potential OSCC therapy.

## RESULTS

### Knocking down Mcl-1 sensitizes human OSCC cells to ABT-737-induced cell death

**Figure 1:**
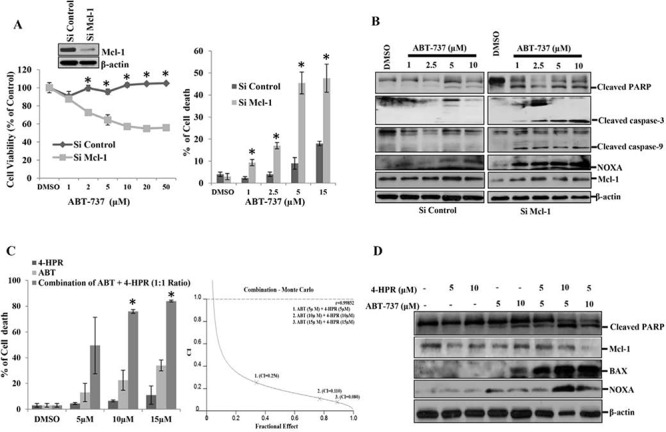
Mcl-1 plays a major role in the carcinogenesis of oral squamous cell carcinomas **A. Left panel**: H357 cells were transfected with either siControl or siMcl-1 for 24 hours followed by treatment with the indicated concentrations of ABT-737 for 8 hours, after which MTT assays were performed to measure cell viability. Bars S.D. (*n* = 3, *P < 0.05 vs. Si Control). **Insert**: Immunoblot analysis of siControl or siMcl-1-transfected H357 cells with indicated antibodies. **Right panel**: H357 cells were transfected with either siControl or siMcl-1. After 24 hours the cells were treated as indicated with ABT-737 for 8 hours and trypan blue dye exclusion assays were performed to measure cell death. Bars S.D. (*n* = 3, *P < 0.05 vs. Si Control), **B.** H357 cells were transfected with either siControl or siMcl-1 for 24 hours followed by treatment with the indicated concentrations of ABT-737 for 8 hours after which equal amounts of cell lysates were subjected to immunoblot analysis using the indicated antibodies. **C. Left panel**: H357 cells were incubated with the indicated amount of 4-HPR for 6 hours followed by treatment with the indicated amount ABT-737 for 24 hours after which cell death was measured using trypan blue dye exclusion assays. The drugs were used at a fixed ratio (HPR: ABT::1:1). Bars SD. (*n* = 3, *P < 0.05 vs. 4-HPR). **Right Panel**: Cells were treated as mentioned in the left panel and the Combination Index (CI) was determined by using CalcuSyn software. Combination Index (CI) values less than 1.0 indicate a synergistic interaction. **D.** H357 cells were treated with the indicated amount of 4-HPR for 6 h followed by treatment with indicated amounts of ABT-737 for 24 h after which equal amount of cell lysates were subjected to immunoblot analysis using the indicated antibodies.

To determine whether Mcl-1 is a key survival factor in OSCC, we knocked down Mcl-1 genetically and with pharmacological inhibitors in the human OSCC line H357 and measured cell death in response to ABT-737. H357 cells transfected with control siRNA (siControl) were not affected even at higher doses of ABT-737 after 8-hour of treatment (Fig. 1A left panel). On the contrary, Mcl-1 siRNA (siMcl-1)-transfected cells showed a significant reduction in cell viability in response to ABT-737. Next, we performed cell death assays at 8-hours post-treatment and found that siMcl- 1-transfected cells showed significantly higher cell death as compared to siControl-transfected cells (Fig. 1A right panel). Furthermore, we analyzed apoptosis markers by immunoblotting and found that siMcl-1-transfected H357 cells showed upregulated NOXA along with enhanced cleaved PARP, caspase-3 and caspage-9 (Fig. 1B). For pharmacological inhibition of Mcl-1, we used 4-HPR (N - (4-Hydroxyphenyl) Retinamide), which downregulates Mcl-1 expression [15]. We evaluated the combinatorial cell death effect of ABT-737 and 4-HPR, where we found 4-HPR and ABT-737 synergistically induced cell death (CI < 1) in H357 cells (Fig. 1C left and right panels). In the combinatorial treatment group, there was increased expression of Bax, NOXA and enhanced PARP cleavage indicating the induction of apoptosis as compared to single agent treatment (Fig. 1D). Overall, our data suggests that OSCC cell survival is dependent on Mcl-1, but not on other anti-apoptotic proteins.

### Mcl-1 antagonist Sabutoclax induces cancer-specific cell death in OSCC

**Figure 2:**
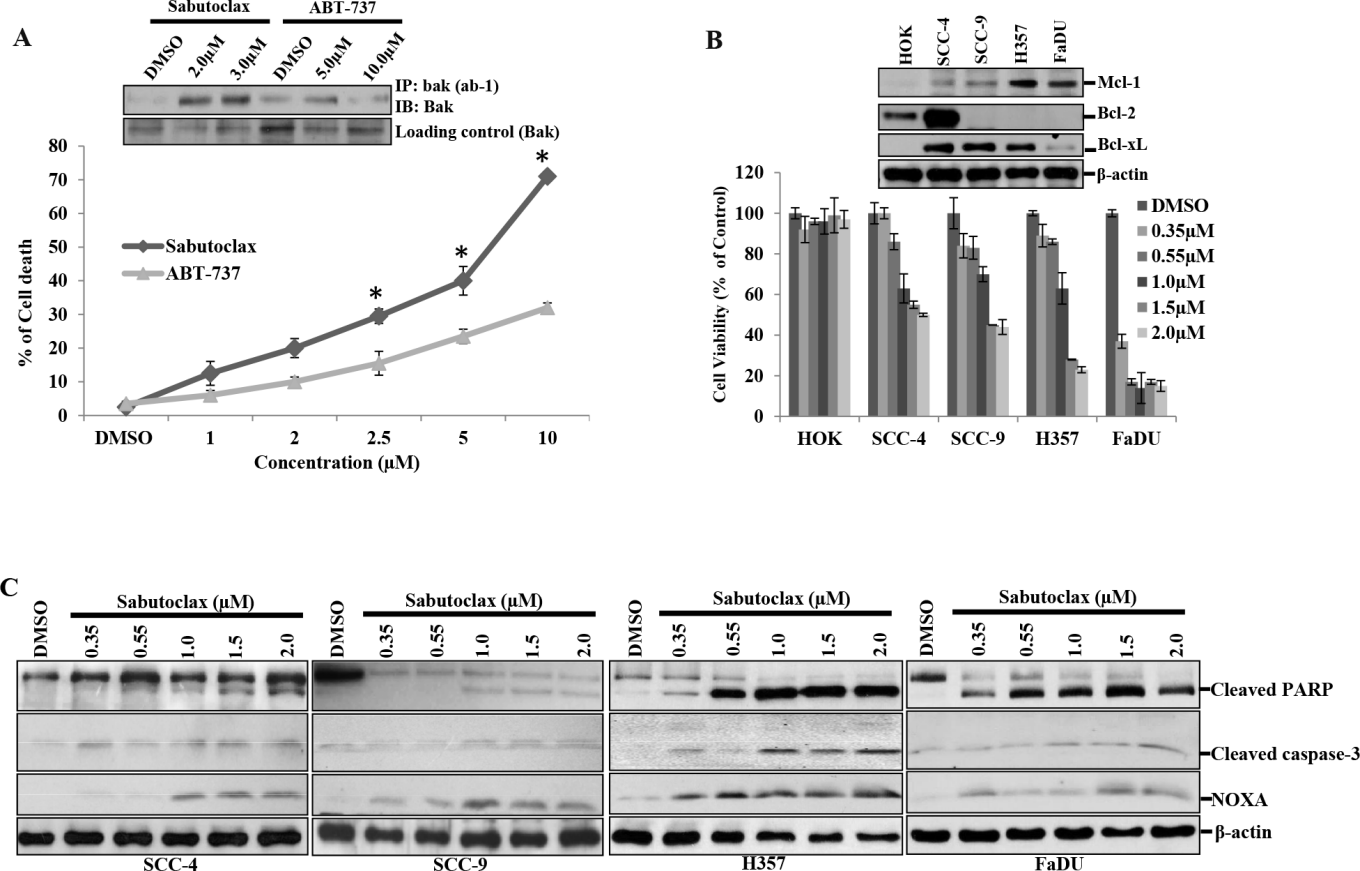
Sabutoclax selectively sensitizes OSCC cells to cell death **A.** H357 cells were treated with the indicated amount of either Sabutoclax or ABT-737 for 48 hours after which cell death was measured using trypan blue dye exclusion assays. Bars S.D. (*n* = 3, *P < 0.05 vs. ABT-737). Insert: H357 cells were treated with the indicated concentration of either Sabutoclax or ABT-737 for 48 hours. Equal amount of cell lysates were subjected to immunoprecipitation with Anti-Bak (ab-1), a clone that specifically recognizes activated Bak after which immunoblotting was performed with a full length Bak antibody to detect activated Bak. **B.** HOK, SCC-4, SCC-9, H357 and FaDU were treated with the indicated amount of Sabutoclax for 48 hours after which cell viability was analyzed by MTT assay. Bars S.D. (*n* = 3). **Insert**: Cell lysates were collected from the indicated cells and immunoblotting was performed using the indicated antibodies. **C.** SCC-4, SCC-9, H357 and FaDU cells were treated with Sabutoclax in a dose-dependent manner for 48 hours after which equal amounts of cell lysates were subjected to immunoblotting with the indicated antibodies.

Assuming that Mcl-1 could function as a principal survival protein in OSCC; we treated human OSCC H357 cells with the Mcl-1 antagonist Sabutoclax or ABT-737 in a dose-dependent manner for 48 hours and determined cell death. We observed that Sabutoclax induced cell death at a much lower dose as compared to ABT-737 (Fig. 2A) in H357 cells. We also found increased levels of activated Bak with Sabutoclax treatment as compared to ABT-737 treatment (insert Fig. 2A). This is important as Bak remains bound to Mcl-1, which prevents its pro-apoptotic actions. Next we analyzed the expression pattern of the anti-apoptotic proteins in a panel of human OSCC lines (SCC-4, SCC-9 and H357) and their normal counterpart HOK. FaDU is a human oropharynx SCC cell line. All the OSCC cells and FaDu expressed elevated amounts of Mcl-1 as compared to HOK, although SCC-4 and SCC-9 showed low levels of Mcl-1 expression as compared to FaDu and H357. Interestingly, with the exception of SCC-4, most SCC cells showed negligible expression of Bcl-2 (Fig. 2B insert). Additionally, dose-dependent cell viability assays were performed with all of the cell lines after treatment with Sabutoclax for 48 hours. The data suggest that Sabutoclax-induced cancer-specific reduction in cell viability occurs in a Mcl-1-dependent manner (Fig. 2B). FaDU and H357 cells, which have the highest levels of Mcl-1, also showed the greatest sensitivity to Sabutoclax. Interestingly, we found that FaDu cells have slightly less Mcl-1 expression as compared to H357 and were more responsive to Sabutoclax. This might be due to barely detectable Bcl-x_L_ expression in FaDu cells (Fig. 2B insert). HOK, whose basal expression of Mcl-1 was low, was found to be least sensitive to Sabutoclax. Immunoblotting was performed to study dose-dependent effects of Sabutoclax on induction of intrinsic apoptosis in SCC-4, SCC-9, H357 and FaDU cells. Sabutoclax treatment resulted in increased expression of NOXA along with enhanced cleavage of PARP and caspase-3 in all cell lines (Fig. 2C).

### Sabutoclax induces selective degradation of mitochondria by autophagy

**Figure 3:**
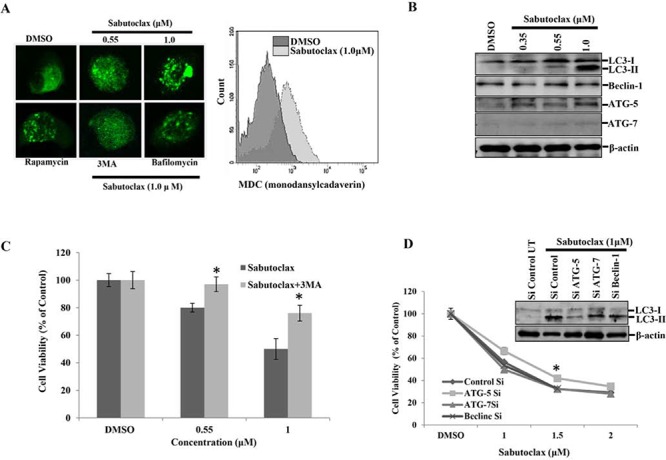
Mcl-1 inhibitor Sabutoclax induces autophagy in OSCC cells **A. Left panel**: H357 cells were transfected with LC3-GFP for 24 hours in a 8 chamber culture slide and treated with the indicated concentration of Sabutoclax, after which the cells were fixed in paraformaldehyde (4%) and confocal microscopy performed to detect GFP (autophagosome punctate structures). Rapamycin (0.1 μM) was used as a positive control for inducing autophagy. 3MA (10 mM) and Bafilomycin (0.2 μM) were used as autophagy inhibitors for this experiment. **Right panel**: H357 cells were treated with 1 μM of Sabutoclax for 48 hours followed by staining with MDC (30 μM) and analyzed by flow cytometry for detection of autophagolysosome vacuoles. **B.** H357 cells were treated with Sabutoclax in a dose-dependent manner for 48 hours after which equal amounts of cell lysates were subjected to immunoblotting with the indicated antibodies. **C.** H357 cells were treated with the indicated concentration of either Sabutoclax or Sabutoclax with 3MA (10 mM) for 48 hours, after which cell viability was measured by MTT assay. Bars S.D. (*n* = 3, *P < 0.05 vs Sabutoclax). **D.** H357 cells were transfected with the indicated siRNAs for 24 hours followed by treatment with the indicated concentrations of Sabutoclax for 48 hours. Cell viability was measured by MTT assay. Bars S.D. (*n* = 3, *P < 0.05 vs. Control Si). **Insert**: H357 cells were transfected with indicated siRNAs for 24 hours followed by treatment with 1 μM Sabutoclax for 48 hours, after which immunoblot analyses was performed with the indicated antibodies.

It has been established previously that Mcl-1 inactivation leads to induction of autophagy [16]. Therefore, we wanted to determine if Sabutoclax could induce autophagy in OSCC. Sabutoclax treatment in H357 cells resulted in the formation LC3-GFP punctate staining as demonstrated via confocal microscopy (Fig. 3A left panel). We also performed flow cytometry with H357 and found that MDC staining was more intense in Sabutoclax-treated H357 cells, confirming that Sabutoclax induces the formation of autophagolysosome vacuoles in OSCC cells (Fig. 3A right panel). Next, we performed immunoblotting for autophagy markers to support these observations, where we found that treatment of H357 cells with Sabutoclax resulted in an increased conversion of LC3-I to LC3-II and upregulated ATG-5 expression (Fig. 3B). Interestingly, autophagy inhibition by 3MA could protect H357 cells from Sabutoclax-induced reduction of cell viability (Fig. 3C). Similarly, when ATG-5 was knocked down using siRNA in H357 cells, Sabutoclax-induced LC3-I to LC3-II conversion was inhibited. Additionally, knock down of ATG-5 rescued the Sabutoclax-induced reduction of cell viability. These data confirm a role of ATG-5 in Sabutoclax-induced autophagy (Fig. 3D).

**Figure 4:**
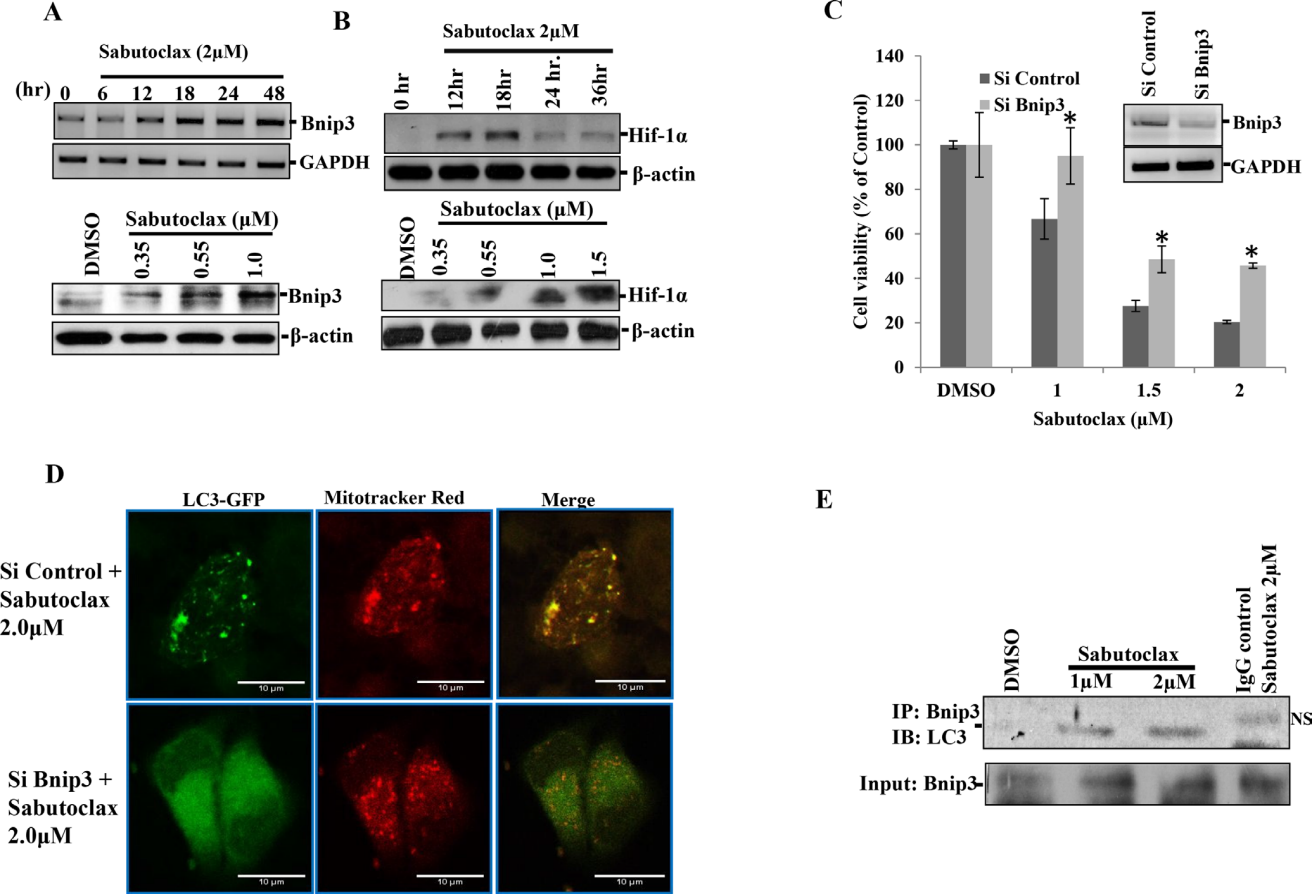
Sabutoclax induces Bnip3-mediated mitophagy in OSCC **A. Upper panel**: H357 cells were treated with Sabutoclax (2 μM) for the indicated time points, RNAs were isolated and mRNA expression of the indicated genes were determined by RT-PCR. **Lower panel**: H357 cells were treated with the indicated amount of Sabutoclax for 48 hours after which immunoblotting was performed with the indicated antibodies. **B. Upper panel**: H357 cells were treated with Sabutoclax (2 μM) for the indicated time points and lysates were collected to perform western blotting with the indicated antibodies. **Lower panel**: H357 cells were treated with the indicated amount of Sabutoclax for 18 hours after which immunoblotting was performed with the indicated antibodies. **C.** H357 cells were transfected with either siControl or siBnip3 for 24 hours followed by treatment with the indicated concentration of Sabutoclax for 48 hours, after which MTT assays were performed to measure cell viability. Bars S.D. (*n* = 3, *P < 0.05 vs. Si Control). Insert; H357 cells were transfected with either siControl or siBnip3 for 24 hours after which RNAs were isolated and RT-PCR was performed for Bnip3 and GAPDH. **D.** H357 cells were transfected with siBnip3 or siControl and co-transfected with LC3-GFP and treated with 2 μM of Sabutoclax for 48 hours. The cells were stained with CM-Rox Mitotraker red (30 nM) for 30 minutes after which confocal microscopy was done to detect GFP and mitotraker red. **E.** H357 cells were treated with the indicated amount of Sabutoclax for 48 hours. Equal amounts of cell lysates were subjected to immunoprecipitation with Bnip3 and immunoblotted for LC3 (NS: nonspecific band).

To investigate the physiological role of autophagy in Sabutoclax-induced toxicity, a human autophagy PCR array was evaluated (SA Bioscience) using mock- and Sabutoclax-treated FaDU cells (Supplementary Fig. S1). Results showed significant upregulation of Bnip3, a Bcl-2 family protein that is considered to have pro-apoptotic activity and that is a Hif-1α-induced gene [17]. Interestingly, it is reported that Bnip3 induces mitophagy as its N-terminal domain hooks itself to the mitochondria, whereas its C-terminal domain has an LC3 interacting region (LIR) that binds to LC3 in autophagosomes [18]. We confirmed our PCR array data in H357 cells, which showed that Sabutoclax upregulated Bnip3 and Hif-1α in both a time- and dose-dependent manner (Fig. 4A & 4B). Additionally, knocking down Bnip3 could rescue H357 cells from Sabutoclax-induced reduction of cell viability (Fig. 4C). Next, we performed confocal microscopy to detect mitophagy in Sabutoclax-treated H357 cells. Results showed that mitophagy induction was observed in the siControl-transfected group, which was not evident in the siBnip3-transfected group (Fig. 4D). In this experiment, all groups were co-transfected with LC3-GFP and siBnip3 followed by treatment with Sabutoclax. At the end of the experiment the mitochondria were stained with CM-ROX mitotracker red. The Sabutoclax-treated siControl group showed a significant level of mitophagy (the yellow dots indicate mitophagy), whereas siBnip3-transfected cells showed reduced mitophagy. Furthermore, our immunoprecipitation data suggest that treatment of H357 cells with Sabutoclax leads to the interaction of Bnip3 with LC3 thus inducing Bnip3-mediated mitophagy (Fig. 4E).

### Mcl-1 antagonist inhibits tumor growth in a carcinogen-induced tongue OSCC mouse model

**Figure 5:**
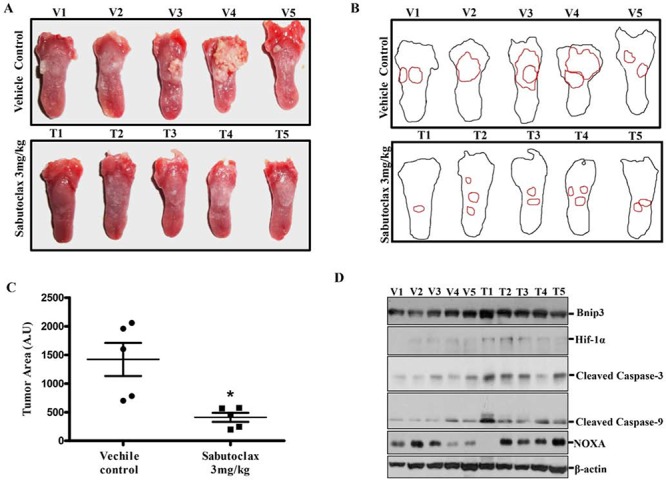
Sabutoclax inhibits tumor growth in a carcinogen-induced tongue OSCC mouse model **A.** BALB/c mice were subjected to oral administration of 4NQO (50 μg/ml) for 20 weeks after which the mice were reverted back to normal water for 4 weeks. After 24 weeks, when visible tumors appeared on the tongue, mice were divided into 2 groups, i.e., vehicle control group (*n* = 5, V1 to V5) and Sabutoclax-treated group (*n* = 5, T1 to T5) where Sabutoclax was given at a concentration of 3 mg/kg body weight. The drugs were injected I.P. twice a week for 6 weeks. At the end of the experiment the mice were sacrificed and the tongues were photographed. **B.** Digital outline of each representative tongue from control and Sabutoclax-treated mice as described in panel A. Red lining indicate tumor burden in the respective tongue. **C.** Tumor areas as indicated in 5B were quantified by ImageJ software in arbitrary units. In scatter plots single scatter indicated individual mouse tumor area. Statistical significance of average tumor area was analyzed by Graph Pad Software (statistical significance *P < 0.05, student *t*-test where *n* = 5). **D.** Cell lysates were isolated from tongue tumors and equal amounts of protein were subjected to immunoblot analysis with the indicated antibodies with β-actin used as a loading control. V indicates solvent DMSO control-treated mice and T indicates Sabutoclax-treated mice.

To evaluate if Sabutoclax could inhibit OSCC tumor growth *in vivo*, we established a 4-NQO-induced tongue OSCC model in BALB/c mice. 4-NQO (50 μg/ml) was administered in drinking water for 20 weeks after which regular water was provided for four weeks. After visible tumor formation in the tongue at 24 weeks, mice were injected with vector control or 3 mg/kg Sabutoclax I.P. twice a week for six weeks. Sabutoclax significantly inhibited tumor growth in the tongue of 4-NQO-treated BALB/c mice (Fig. 5A, 5B & 5C). Immunoblotting using lysates derived from these tumors showed that Bnip3 and Hif-1α were marginally elevated in several Sabutoclax-treated tongue tumors (Fig. 5D). In addition, we found increased expression of NOXA, cleaved caspase-3 and cleaved caspase-9 were upregulated in several of the Sabutoclax-treated tumors indicating induction of apoptosis.

### Sabutoclax and Celecoxib synergistically inhibit the growth of OSCC

**Figure 6:**
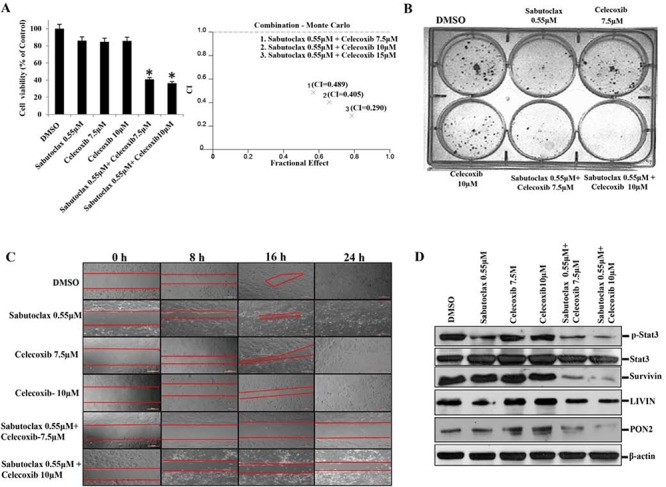
The combination regimen of Sabutoclax and Celecoxib inhibits the growth of OSCC **A. Left Panel**: H357 cells were treated with either Sabutoclax or Celecoxib, alone or in combination, for 48 hours after which cell viability was measured using MTT assays. Bars S.D. (*n* = 3, *P < 0.05 vs. DMSO). **Right Panel**: Cells were treated as indicated in the left panel and Combination Index (CI) was determined by using CalcuSyn software. Combination Index (CI) values less than 1.0 indicate a synergistic interaction. **B.** H357 cells (400 cells/well) were seeded in 6-well culture plates. 24 hours after cell seeding the indicated concentrations of drugs were added alone or in combination and cells were incubated for 18 days after that they were stained with 0.5% crystal violate solution and photographed. **C.** H357 cells were seeded at a density of 5 × 10^5^ cells per well in 24-well flat-bottom culture plates. After reaching 90% confluence wounds were created using a pipette tip. Cells were treated with the indicated concentrations of either Sabutoclax or Celecoxib alone or in combination. Bright field images were captured at time points (*t* = 0, 8, 16 and 24 hours). **D.** H357 cells were treated with the indicated amount of Sabutoclax and Celecoxib alone or in combination for 48 hours after which equal amounts of cell lysates were subjected to immunoblotting with the indicated antibodies.

**Figure 7:**
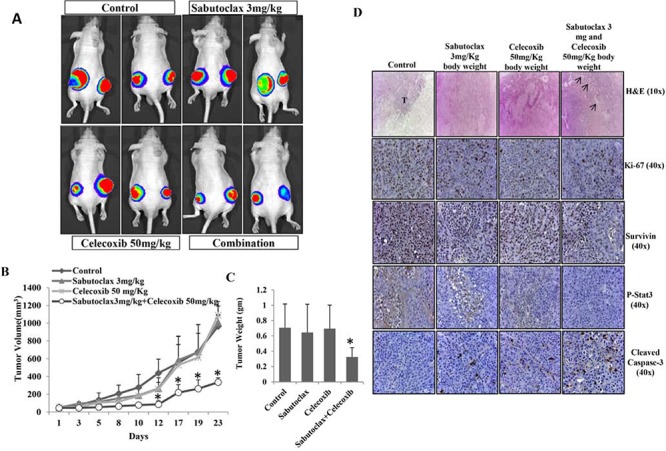
The combination regimen of Sabutoclax and Celecoxib inhibits OSCC tumor growth in athymic nude mice **A.** Athymic nude mice bearing FaDU-Luc xenograft in both right and left flanks were treated with Vehicle, Sabutoclax alone (3 mg/kg), Celecoxib alone (50 mg/kg) or a combination of Sabutoclax (3 mg/kg) and Celecoxib (50 mg/kg). Tumor growth was visualized by BLI using a Xenogen imaging system (out of eight, 2 representative mice from each group are displayed). **B.** Tumor growth was measured using slide calipers and plotted as a graph. Bars S.D. (*n* = 8, *P < 0.05 vs. Control). **C.** At the end of the experiment the mice were sacrificed and tumor weight was measured. Bars S.D. (*n* = 8, *P < 0.05 vs. Control). **D.** At the end of the study in *A*, tumors were harvested and immunohistochemistry was performed for Survivin, Ki-67, p-Stat3, cleaved caspase-3.

All of the above studies showed that Sabutoclax as a single agent could inhibit the growth of OSCC. However, single agent treatment of cancer is unrealistic due to the complex nature of the disease. The development of novel combination therapies is critical to successfully eradicate human cancer. Due to a large basis of literature supporting the role of COX-2 in OSCC development and progression [19, 20], we chose to evaluate a COX-2 inhibitor, Celecoxib, in combination with Sabutoclax. *In vitro*, Sabutoclax and Celecoxib synergistically inhibited the growth of H357 cells, which was demonstrated using cell viability, colony forming and scratch assays (Fig. 6A, 6B & 6C). Immunoblotting data showed that the combination treatment inhibited cell proliferation markers such as Survivin, Stat-3, LIVIN and PON2 (Figure 6D). Additionally, to investigate if this combinatorial effect could be recapitulated *in vivo*, we performed experiments in a nude mouse xenograft model using the human SCC cell line FaDU. As was evident from the bioluminescent imaging (BLI) data shown in Fig. 7A, the combination of sublethal doses of Sabutoclax (3 mg/kg) and Celecoxib (50 mg/kg) significantly inhibited tumor growth and size as compared to the single agent-treated groups (Fig. 7B & 7C). Immunohistochemistry also demonstrated that in the combination group there was a significant decrease in cell proliferation signals like Ki-67, Survivin, and p-Stat3. Additionally, we found increased expression of cleaved capase-3 in combination group indicating induction of apoptosis (Fig. 7D).

## DISCUSSION

**Figure 8:**
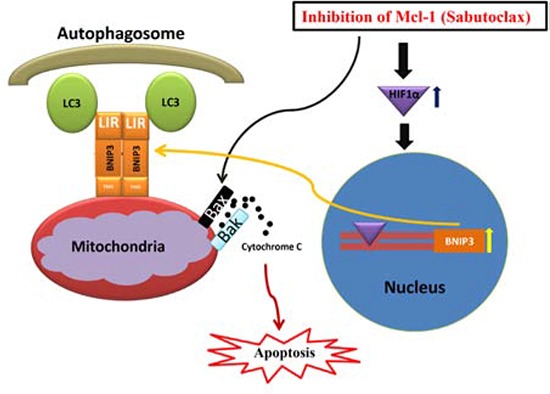
Hypothetical model of action for the anti-tumor activity of Mcl-1 antagonist Sabutoclax in OSCC cells Inhibition of Mcl-1 by the BH3 mimetic Sabutoclax in OSCC activates pro-apoptotic proteins that result in release of cytochrome c into the cytoplasm, which triggers apoptosis. In addition, Sabutoclax triggers Hif-1α induced Bnip3 up regulation in OSCC cells. Bnip3 binds to mitochondria through its N-terminal domain and interacts with LC3 of the autophagosome through LC3 interacting region in the C-terminus to cause mitophagy, which also contributes to Sabutoclax-mediated cell death in OSCC.

BH3 mimetic ABT-737 binds efficiently to Bcl-2, Bcl-x_L_ and Bcl-w, but shows limited affinity towards Mcl-1. Preclinical animal studies have suggested that as a single agent ABT-737 exerts excellent anti-tumor activity in hematological cancers [21], but most solid cancers including prostate, breast and colon carcinomas demonstrate innate resistance against ABT-737 [22–24]. These available reports suggest that hematological cancer cell survival is Bcl-2-dependent, whereas solid cancers typically depend more on Mcl-1 for their survival. In the present study, OSCC showed resistance towards ABT-737, whereas Mcl-1 antagonist Sabutoclax induced profound cell death even at low doses. These data suggest that OSCC cell survival is dependent upon Mcl-1, but not on Bcl-2 or Bcl-x_L_. In addition, we found that the majority of human OSCC cell lines express elevated amounts of Mcl-1, but negligible amounts of Bcl-2, a phenomenon recently observed in HNSCC by independent researchers [14, 25]. Additionally, immunoblotting was performed to determine the expression of Bcl-2, Bcl-x_L_ and Mcl-1 in eleven different OSCC patient tumors. All 11 patient tumors expressed Mcl-1, but only 5 out of 11 patient tumors expressed Bcl-2 at negligible levels (Supplementary Fig. 2). These data indicate the dependence of OSCC tumor survival on Mcl-1. We found that Sabutoclax induced cancer-specific cell death in OSCC in a Mcl-1-dependent manner, which was attributed to both Bak-induced apoptosis and Bnip3-mediated mitophagy (Fig. 8). This finding was confirmed by our observation that the pan-caspase-inhibitor z-VAD-FMK rescued H357 cells from Sabutoclax-induced reduction of cell viability (Supplementary Fig. 3A). Additionally, when Bnip3 was knocked down, the Sabutoclax-induced loss of mitochondrial membrane potential was not observed indicating cytotoxic activation of mitophagy (Supplementary Fig. 3B).

Treatment of OSCC cells with Sabutoclax resulted in accumulation / up regulation of Hif-1α even in a normoxic environment, which in turn induced Bnip3-mediated mitophagy. It was previously established that Hif-1α is a transcriptional regulator of Bnip3 [26]. Recently, AT-101 (modified enantiomer of gossypol) was shown to stabilize Hif-1α protein without any increase in mRNA levels [27]. Furthermore, under normoxic conditions Hif-1α is spontaneously hydroxylated by prolyl hydroxylase, an enzyme that requires oxygen and iron to perform its enzymatic activity. AT-101 treatment caused intracellular iron chelation and resulted in accumulation of Hif-1α in nerve sheath tumor cells [27]. In our study, Sabutoclax-induced Hif-1α, Bnip3 and LC3-II and expression was blocked significantly when the media was supplemented with iron in the form of ferric citrate (Supplementary Fig. 4A). Similarly, Sabutoclax-induced reduction of cell viability in OSCC was rescued by treatment with ferric citrate (Supplementary Fig. 4B). Therefore, it is possible that Sabutoclax, being an Apogossypol derivative, may also chelate iron in OSCC cells.

COX-2 expression is elevated in various cancers including head and neck, breast, gastric, hepatocellular and colorectal carcinomas [19, 28–31]. Additionally, COX-2 activation is an early event in OSCC and is overexpressed in almost 80% of oral cancer cases [20]. Aberrant regulation of cyclooxygenase-2 (COX-2) results in an increased abundance of its principal metabolic product, prostaglandin E2, which can affect multiple aspects of cancer development and progression. COX-2 activation leads to the evasion of apoptosis, angiogenesis and metastasis [28]. Recently, COX-2 activation has been linked with the development of radioresistance [28]. Considering that combination therapy may be the most effective strategy of combating cancer, we decided to evaluate the combinatorial effect of Sabutoclax with Celecoxib, a COX-2 inhibitor, both *in vitro* and *in vivo*. Our data indicate that the combination of Sabutoclax and Celecoxib inhibits proliferation signals like Survivin, p-Stat3, PON2 and LIVIN. PON2 lowers the stress-induced pro-apoptotic protein CHOP, which contributes to the development of chemotherapeutic resistance in cancer cells [32]. Livin is a member of the inhibitor of apoptosis protein (IAP) family, which may sequester SMAC, preventing it from antagonizing XIAP-mediated inhibition of caspases (livin). We also found that this novel combination showed significantly increased inhibition of tumor growth *in vivo* as compared to either agent used alone.

Mcl-1 is critical for survival of regulatory T cells (T_reg_) [33]. Mice having T_reg_-specific deletion of Mcl-1 lose T_reg_ cells resulting in autoimmunity [33]. Since the anti-tumor efficacy of Sabutoclax was evaluated in a carcinogen-induced OSCC model in BALB/c mice (Figure 5), it was important to determine the potential impact of Sabutoclax on T lymphocytes in BALB/c mice. To address this issue, we performed additional animal studies where BALB/c animals were treated with vehicle control or Sabutoclax (1 mg/kg and 3 mg/kg body weight) IP twice a week for 6 weeks. At the end of the experiment, all animals were sacrificed and peripheral blood, spleen and lymph nodes were collected and subjected to flow cytometry analysis to detect T_reg_ cell populations (CD3^+^CD4^+^ FoxP3^+^). As evident in Supplementary Figure 5 we did not find any significant difference in T_reg_ cell populations between vehicle control- and Sabutoclax-treated mice. In addition, we also did not find any significant difference between CD8^+^ cell populations between vehicle control- and Sabutoclax-treated mice (Supplementary figure 6). These evidences indicate that Sabutoclax has a minimal impact on T_reg_ cell populations. Although Sabutoclax inhibits Mcl-1, it may not mimic the complete knock out of Mcl-1 in the T_reg_ cell populations.

In conclusion, OSCC showed resistance to Bcl-2 antagonist ABT-737, supporting the concept that OSCC cell survival is dependent upon Mcl-1. The BH3 mimetic Sabutoclax, which significantly targets Mcl-1 in addition to the other anti-apoptotic Bcl-2 proteins, induced cancer-specific cell death in OSCC alone or in combination with Celecoxib. Considering the importance of Mcl-1 in OSCC survival, our future studies will focus on elucidating the potential role of Mcl-1 in chemo- and radioresistance of OSCC. Overall, this study provides important evidence that highlights Mcl-1 as a potentially viable therapeutic target in OSCC and also presents evidence of efficacy of a novel and exciting combination therapy for this disease.

## MATERIALS AND METHODS

### Cell lines and culture conditions

Human OSCC cell lines H357, SCC-4 and SCC-9 were obtained from Sigma-Aldrich (collected from European Collection of Cell Cultures). The human pharynx squamous cell carcinoma cell line FaDU was obtained from the American Type Culture Collection. SCC-4 and SCC-9 cell lines were cultured in Dulbecco's Modified Eagle's Medium/F12 (DMEM/F12; Life Technologies) supplemented with 10% Fetal Bovine Serum (FBS), 0.4 μg/ml hydrocortisone (Sigma-Aldrich) and 0.5 mM sodium pyruvate (Life Technologies). FaDU was cultured in Eagle's Minimum Essential Medium (Life Technologies) supplemented with 10% FBS. H357 cells were cultured in DMEM/F12 medium supplemented with 10% FBS and 0.5 μg/ml sodium hydrocortisone succinate (Sigma-Aldrich). Primary Human Oral Keratinocytes (HOK) were isolated from healthy gingival tissue of normal human patients and maintained in keratinocyte serum-free Media (Life Technologies) supplemented with 2% FBS, bovine pituitary extract 60 mg/mL (Life Technologies) and epidermal growth factor (1 ng/mL) (Life Technologies) as described previously [34].

### Reagents

Celecoxib, ABT-737, and z-VAD-FMK were purchased from Santa Cruz Biotechnology. Sabutoclax was synthesized in the laboratory of Dr. Maurizio Pellecchia (Sanford-Burnham Medical Research Institute, La Jolla, CA, USA) (9, 11). Rapamycin, Bafilomycin A1 and 3-Methyladenine (3-MA) were obtained from Sigma-Aldrich.

### Assessment of cell viability and cell death

Cell viability was measured by 3-(4, 5-dimethylthiazol-2-yl)-2, 5-diphenyltetrazolium bromide (MTT; Sigma-Aldrich) assay [35] and cell death was measured by trypan blue dye exclusion assay as described earlier [36].

### Transient transfection and siRNA

Transfection was performed using Lipofectamine 2000 (Life Technologies) according to the manufacturer's protocol with plasmid and siRNAs. Control siRNA (DO-001210-01-05) and Mcl-1 siRNA (M-004501-08) was purchased from Thermo Scientific. Bnip3 siRNA was obtained from Ambion, Life Technologies (4392420). ATG-5 siRNA (Sc-41445), sh-RNA Beclin-1 (SC-29797-Sh) and siRNA ATG-7 (Sc-41447) were purchased from Santa Cruz.

### Semi-quantitative RT-PCR analysis

Total RNAs were isolated by using GE Healthcare RNAspin kit according to the manufacturer's protocol. Equal amount of RNAs were subjected to cDNA synthesis using a reverse transcription core kit from Erogentec. Primers for PCR: GAPDH forward primer 5′-TCGGAGTCAACGGATTTGGT-3′ and reverse primer 5′-TTGCCATGGGTGGAATCATA-3′. Bnip3 primers forward- 5′-CCACCTCGCTCGCAGACACCAC-3′ and reverse primer 5′-GAGAGCAGCAGAGA TG GAAGGAAAAC-3′. Bnip3 and GAPDH were amplified in a PCR reaction using annealing temperatures of 60°C for Bnip3 and 53°C for GAPDH. PCR products were separated on 2% agarose gel and image captured on Chemi XRS Gel Documentation System (Bio-Rad).

### Autophagy PCR array

FaDU cells were treated for 24 hours with either mock or 0.7 μM Sabutoclax. After treatment, total RNA was extracted followed by cDNA production from RNA (1 μg) using the RT2 First Strand Kit from SABiosciences according to the manufacturer's instructions. Human Autophagy PCR arrays (PAHS-084, SABioscience) were run on the ABI Prism 7000 Sequence Detection System (Applied Biosciences). The results were analyzed using software from SABiosciences according to the manufacturer's instructions.

### Immunoprecipitation and immunoblotting

Equal amounts of cell lysates were loaded for immunoprecipitation or immunoblotting using the indicated primary antibodies [37]. Primary antibodies used in this study were as follows: LC3, ATG-5, ATG-7, Caspase-3, Caspase-9 (Novus biological), PARP, p-Stat-3, Survivin, cleaved caspase-3 and Bcl-2 (Cell Signaling Technology), Hif1α, Beclin-1, Bim, Bax (BD Bioscience), Bnip3 (Abcam), Noxa, LIVIN (Imgenex), PON2 (Abnova), β-actin (Sigma-Aldrich). Mcl-1, Bak, Bcl-x_L_ (Santa Cruz). Anti-Bak (Ab-1) were developed against recombinant human Bak from which the C-terminal and transmembrane domains were deleted (Calbiochem).

### Flow cytometry

The autofluorescent agent Monodansylcadaverine (MDC, Sigma-Aldrich) was used to measure the autophagolysosome vacuoles. H357 cells were treated with the indicated concentration of Sabutoclax. After 48 hour of treatment, cells were incubated with 30 μM MDC in DMEM/F12 at 37°C for 20 minutes. Cells were then washed three times with 1X PBS and analyzed by Flow Cytometry (BD LSRFortessa) at λex 338 nm; λem 500 nm in UV laser [38]. Mitochondrial membrane potential was measured by using a cationic dye JC-1 (T3168, Life Technologies), which exhibits potential-dependent accumulation in mitochondria (excitation at 488 nm and emission at 527 nm). H357 cells were transfected with either si-Bnip3 or si-control and treated with the indicated concentration of Sabutoclax for 48 hours. Cells were stained with JC-1 (50 μM) at 37°C for 30 minutes and monomeric form of JC-1 was analyzed by flow cytometer (BD FACSCalibur) [39].

### Monitoring autophagy and mitophagy

LC3-GFP was used to detect autophagy punctate structures. H357 cells were transfected with LC3-GFP for 24 hours in an 8-chamber culture slide (BD Falcon) and exposed to the indicated concentrations of Sabutoclax. Confocal microscopy was used to detect autophagy punctate structures. Rapamycin (0.1 μM) was used as a positive control for inducing autophagy. 3-MA (10 mM) and Bafilomycin (0.2 μM) were used as autophagy inhibitors for this experiment. H357 cells were transfected with si-Bnip3 or si-control and co-transfected with LC3-GFP and treated with the indicated concentrations of Sabutoclax. After 48 hours of treatment, cells were incubated with 30 nM CM-Rox Mitotraker red (Life Technologies) in DMEM/F12 medium at 37°C for 20 minutes. Cells were then washed with 1X PBS and fixed in 4% paraformaldehyde for 15 minutes. Confocal microscopy (LEICA TCS-SP5) was performed to detect GFP and mitotraker red. The yellow dots indicate mitophagy.

### Drug combination studies

In drug combination studies, Sabutoclax was used alone or in combination with Celecoxib and ABT-737 alone or combination with 4-HPR (Sigma Aldrich) to treat H357 cells. Cell viability or cell death was determined by MTT assay or trypan blue exclusion assay, respectively. Combination index was determined by Calcusyn software (Biosoft), using the Chou-Talalay method [40]. Calcusyn combination index CI < 1 indicates synergism.

### *In vivo* animal model

Institutional Animal Ethics Committee of “Institute Life Sciences” approved the protocols followed for conducting experiments on mice. The carcinogen-induced tongue mouse model experiments were performed as described earlier by Czerninski et al. with slight modification [41]. In all experiments, 4 to 6 week-old female BALB/c mice weighing 20–25 g were used and 4NQO (4-Nitroquinoline 1-oxide, Sigma-Aldrich), which serves as a surrogate of tobacco exposure, was used as a carcinogen. Oral administration of 4NQO (50 μg/ml) with drinking water was applied for 20 weeks, after which all animals were reverted back to regular water and monitored for up to 24 weeks. After visible tumors appeared on the tongues of the mice, animals were randomly divided in two groups (*n* = 5 mice / group). One group of mice were injected intraperitoneally (I.P.) with 3 mg / kg body weight Sabutoclax (dissolved in ethanol/Cremophor EL/saline = 10:10:80) and another group of mice were injected I.P. with vehicle control DMSO, twice a week for 6 weeks. After completion of treatment all animals were sacrificed and tongue tissue was collected during necropsy. Whole cell protein was extracted from tongue tumors.

### Assessment of the impact of Sabutoclax on the T lymphocytes of BALB/c mice

For this experiment 8 week-old BALB/c female mice weighing 20–25 g were used. Animals were randomly divided in three groups (*n* = 5 mice per group). One group of mice were injected intraperitonealy (I.P) with 1 mg / kg, a second group with 3mg/kg body weight Sabutoclax (dissolved in ethanol/Cremophor EL/saline = 10:10:80) and a third group of mice were injected I.P with vehicle control DMSO (dissolved in ethanol/Cremophor EL/saline = 10:10:80). The injections were performed twice a week for 6 weeks. After completion of treatment, all animals were sacrificed and peripheral blood, spleen and lymph nodes were collected in medium for further analysis.

Single cell suspension of spleen and lymph nodes were prepared by a sheer force slide dissociation technique. RBCs in peripheral blood and splenic cell suspension were lysed with RBC Lysis buffer (eBioscience). Surface staining was done with anti-mouse CD3-FITC, anti-mouse CD4-BUV395 (BD Bioscience), anti-mouse CD25-PE, anti-mouse CD8-PECy5 (BD Pharmingen) and Anti-Mouse CD4 PE-Cyanine5 (eBioscience). For intracellular staining of FoxP3, samples were fixed by IC-Fixation buffer (eBioscience), permeabilized by permeabilization buffer (eBioscience) according to the manufacture's protocol and stained with anti-mouse FoxP3-Alexafluor647 (BD Bioscience). Stained cells were acquired and analyzed by BD FACS DIVA software in LSR-II flow cytometer (BD Biosciences). Fluorescence minus one controls were used for specific gating.

### Nude mice xenograft model

FaDU-Luc (FaDU stably transfected with Luciferase) cells (2 × 10^6^) were used to establish bilateral subcutaneous tumors on both flanks of 8–10 week old male athymic nude mice. Studies were performed as described earlier [10]. Treatment began when tumors reached ~100-mm^3^. Sabutoclax was dissolved in a 10:10:80 solution of 100% ethanol:Cremophor:PBS and administered at a dose of 3 mg/kg. Celecoxib was dissolved in PBS and administered at a dose of 50 mg/kg. Both drugs were given via I.P. injection three times per week (*n* = 5 mice / group) for four weeks. For *in vivo* imaging of tumors, the mice were anesthetized and injected I.P. with 150 mg/kg luciferin and light emitted from each tumor was determined using a Xenogen system with CCD camera with an integration time of 1 minute. Luminescence measurements were made using Living Image software (version 2.50.1; Xenogen). At the end of the experiment, animals were sacrificed and the tumors were removed and the weight was measured.

### OSCC sample collection and protein isolation

Eleven surgically resected primary OSCC tumors were collected from the Institute of Dental Sciences, Bhubaneswar, India. Immediately after collection, tissues were washed with 1X PBS and homogenized with 0.3 ml RIPA lysis buffer (containing protease inhibitor cocktail) by a homogenizer (Omni-prep, Omni Inc., USA) for 60 seconds at 8000 rpm. The lysates were centrifuged at 10, 000 rpm for 20 minutes at 4°C and supernatants were collected and stored at −80°C. Human Ethics Committee (HEC) of the Institution of Life Sciences approved this study, and informed consent was obtained from all patients.

### Wound-healing

H357 cells were seeded at a density of 5 × 10^5^ cells per well in a 24-well flat-bottom culture plates (BD Falcon). When cells reached 90% confluence, wounds were created using p-10 pipette tips and washed 3X with 1X PBS. Cells were incubated with the indicated concentration of drugs in DMEM/F12 medium supplemented with 10% FBS at 37°C. Images at zero time point (*t* = 0 hour) were captured to record the initial area of the wounds, and recovery images of the wounded monolayers were captured at 8 hour time intervals for 24 hours, when complete healing of the wound area was found in one of the groups. Images were captured in LEICA DMIL microscope and processed by LEICA LAS V4.2 software.

### Clonogenic assay

H357 cells (400 cells/well) were seeded in a 6-well culture plate. 24 hours after cell seeding, the indicated concentration of drugs were added to their respective wells and incubated for 18 days in DMEM/F12 medium supplemented with 10% FBS at 37°C. Drugs and medium were changed every 2 days. After 18 days, visible colonies were stained with 0.5% crystal violet and images captured in Chemi XRS Gel Documentation System (Bio-Rad).

### Statistical Analysis

Data are represented as the mean ± S.D. and analyzed for statistical significance using two-way ANOVA followed by Newman-Keuls test as a post hoc test. *P* < 0.05 was considered as statistically significant.

## SUPPLEMENTARY FIGURES


