# Supplementary material for: Mcl-1 is an important therapeutic target for oral squamous cell carcinomas
Source: Oncotarget. 2015 May 14;6(18):16623–37. doi: 10.18632/oncotarget.3932 (PMC4599294; doi:10.18632/oncotarget.3932)
Supplement: Supplementary file 1 [file oncotarget-06-16623-s001.pdf]

SUPPLEMENTARY FIGURES

A

Well	Symbol	Fold Regulation	Comments
B05	ATG9B	2.0307	OKAY
B10	BCL2L1	2.117	OKAY
B11	BECN1	-1.238	OKAY
B12	BID	-2.8245	OKAY
C01	BNIP3	9.3957	OKAY
C02	CLN3	-2.4083	OKAY
C03	CTSB	-3.3823	OKAY
C04	CTSS	-1.6335	OKAY
C05	CXCR4	2.2222	OKAY
C06	DAPK1	2.1465	OKAY
C07	EIF4G1	-2.2784	OKAY
C08	ESR1	1.2675	OKAY
C09	GABARAPL1	3.4153	OKAY
C10	HSP90AA1	-2.3262	OKAY
C11	HSPA8	-2.9039	OKAY
C12	HTT	-1.8125	OKAY
D01	IFNA2	2.0734	OKAY
D02	IGF1	2.2532	OKAY
D03	INS	2.2377	OKAY
D04	MAP1LC3A	4.1182	OKAY
D05	MAP1LC3B	5.6256	OKAY
D06	PRKAA2	2.3327	OKAY
D07	RAB24	2.4318	OKAY
D08	SNCA	-3.6503	OKAY
D09	SQSTM1	2.8719	OKAY
D10	FAM176A	2.5527	OKAY
D11	TNFSF10	-15.116	OKAY
D12	TP53	-2.0392	OKAY
E01	ULK1	2.8324	OKAY

Cat: PAHS-084, SABioscience

B

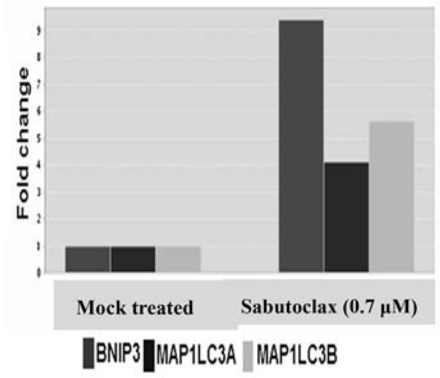

Supplementary Figure S1: Sabutoclax upregulates Bnip3 in human SCC cells. FaDU cells were mock treated or exposed to Sabutoclax (0.7 µM) for 24 hours after which RNA was isolated for testing in a human autophagy PCR array (SABioscience catalog no: PAHS-084). An autophagy PCR array was performed as described by the manufacturer. **A.** Fold-regulation of genes in the autophagy PCR array in Sabutoclax-treated FaDU cells as compared to untreated cells. **B.** Graphical representation of fold up-regulation of Bnip3, MAPLC3A and MAPLC3B in Sabutoclax-treated FaDU cells as compared to Mock-treated cells.

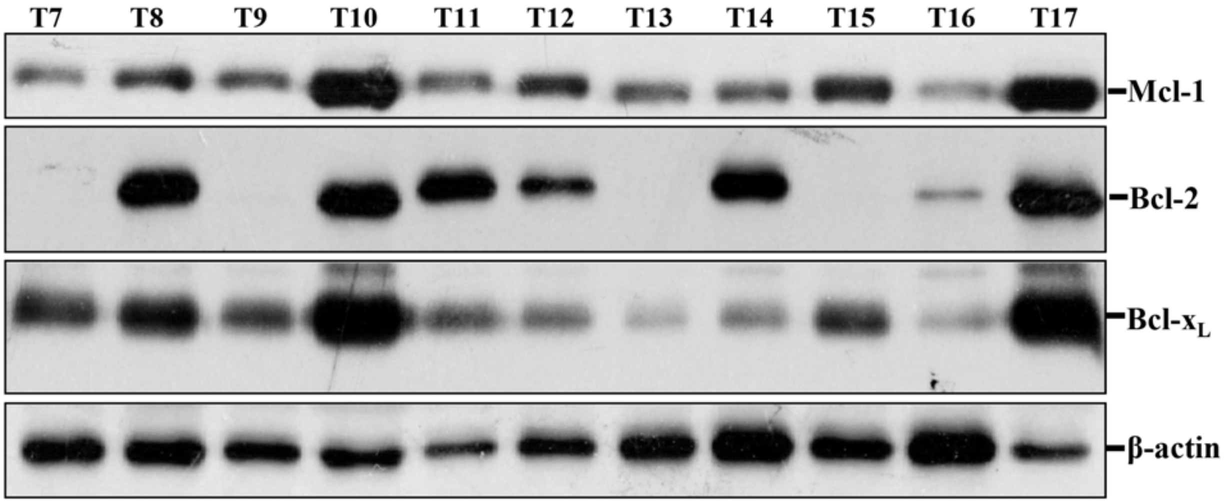

Supplementary Figure S2: Evaluation of anti-apoptotic Bcl-2 protein expression in OSCC tumor samples. Whole cell protein lysates from eleven OSCC primary tumor samples were subjected to immunoblotting with Mcl-1, Bcl-2 and Bcl-x_L antibodies. β-actin was used as a loading control.

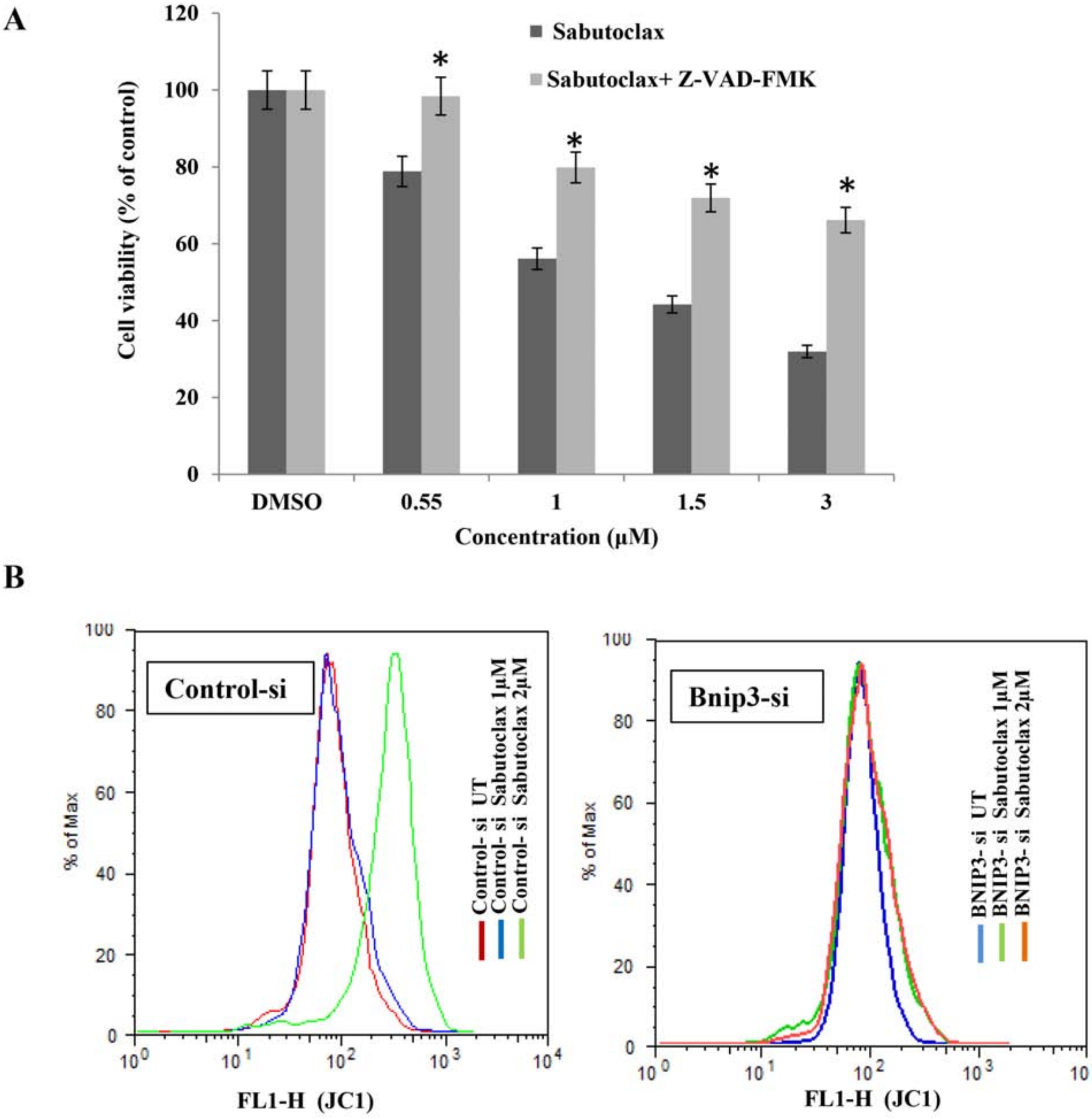

Supplementary Figure S3: Sabutoclax-induced cell death in OSCC is mediated by both apoptosis and toxic mitophagy. A. H357 cells were incubated with DMSO or 50 µM of z-VAD-FMK for 1 hour and then treated with Sabutoclax at the indicated concentration for 48 hours, after which cell viability was measured using an MTT assay. Bars S.D., (n = 3, *P < 0.05 vs Sabutoclax). **B.** H357 cells were transfected with siBnip3 or siControl and treated with the indicated concentration of Sabutoclax for 48 hours. Cells were stained with JC-1 (50 µM) for 30 minutes recommended by the manufacturer and analyzed by flow cytometry as described in Materials and Methods.

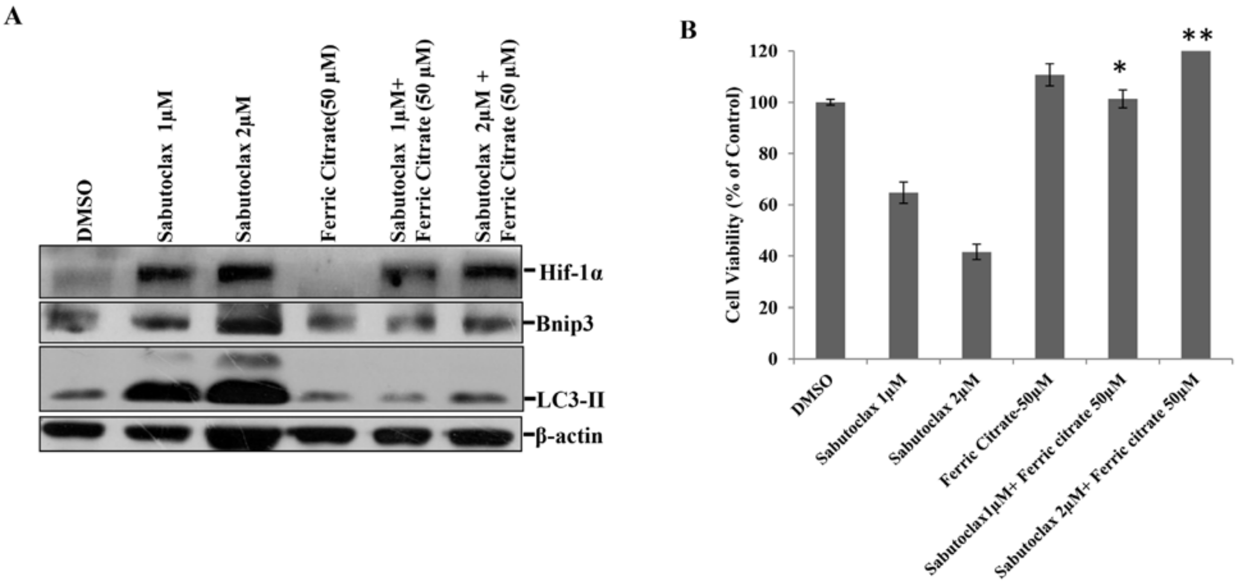

Supplementary Figure S4: Ferric citrate abrogates Sabutoclax-induced cell death in OSCC. A. H357 cells were treated with the indicated concentrations of Sabutoclax and ferric citrate. After 48 hours of treatment total cell lysates were isolated and equal amounts of lysates were subjected to immunoblotting with the indicated antibodies. **B.** H357 cells were treated as described in A and proliferation was determined by MTT assay ($n = 3$, $*P < 0.05$ vs Sabutoclax 1 µM and $**P < 0.05$ vs Sabutoclax 2 µM).

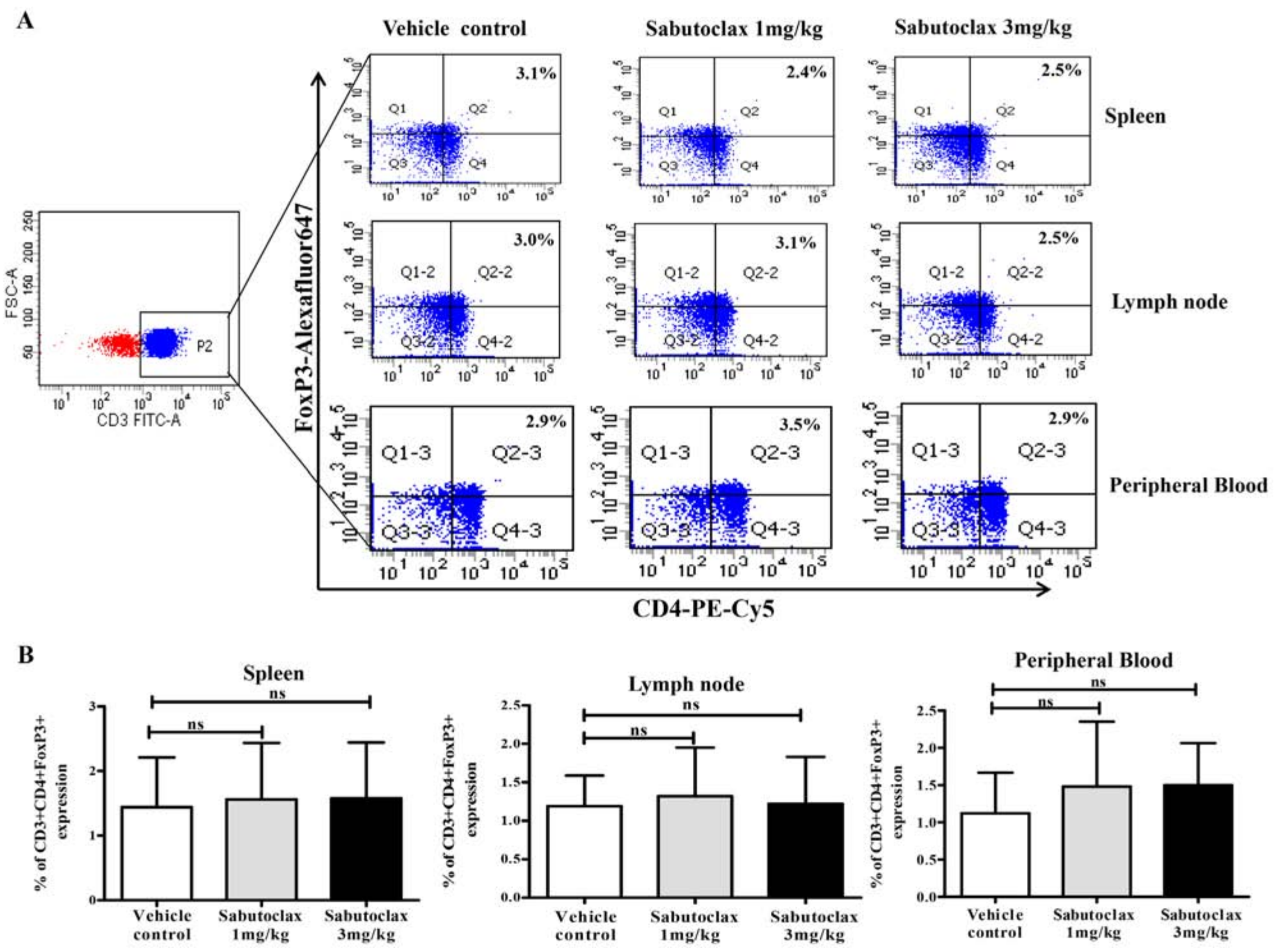

Supplementary Figure S5: Assessment of the impact of Sabutoclax on the Treg of BALB/c mice. BALB/c mice were injected IP with indicated concentration of Sabutoclax twice a week for 6 weeks. After treatment, expression of T_{reg} population **(**$CD3^+CD4^+FoxP3^+$) in peripheral blood, spleen and lymph nodes were analyzed by Flow cytometer as described in materials and methods. **A.** The gating strategy of CD3+ cell population (left) and one representative flow cytometry analysis of $CD3^+CD4^+$ $FoxP3^+$ cell population from each group (right) are indicated. **B.** Bar graphs represent the % of T_{reg} population **(**$CD3^+CD4^+FoxP3^+$) in spleen, lymph node and peripheral blood. Error bars represent SE where $n = 5$ mice per group. Statistical analysis were performed using Graph pad software (one way ANOVA) where $*p < 0.05$ considered as statistical significance. ns- Non significance.

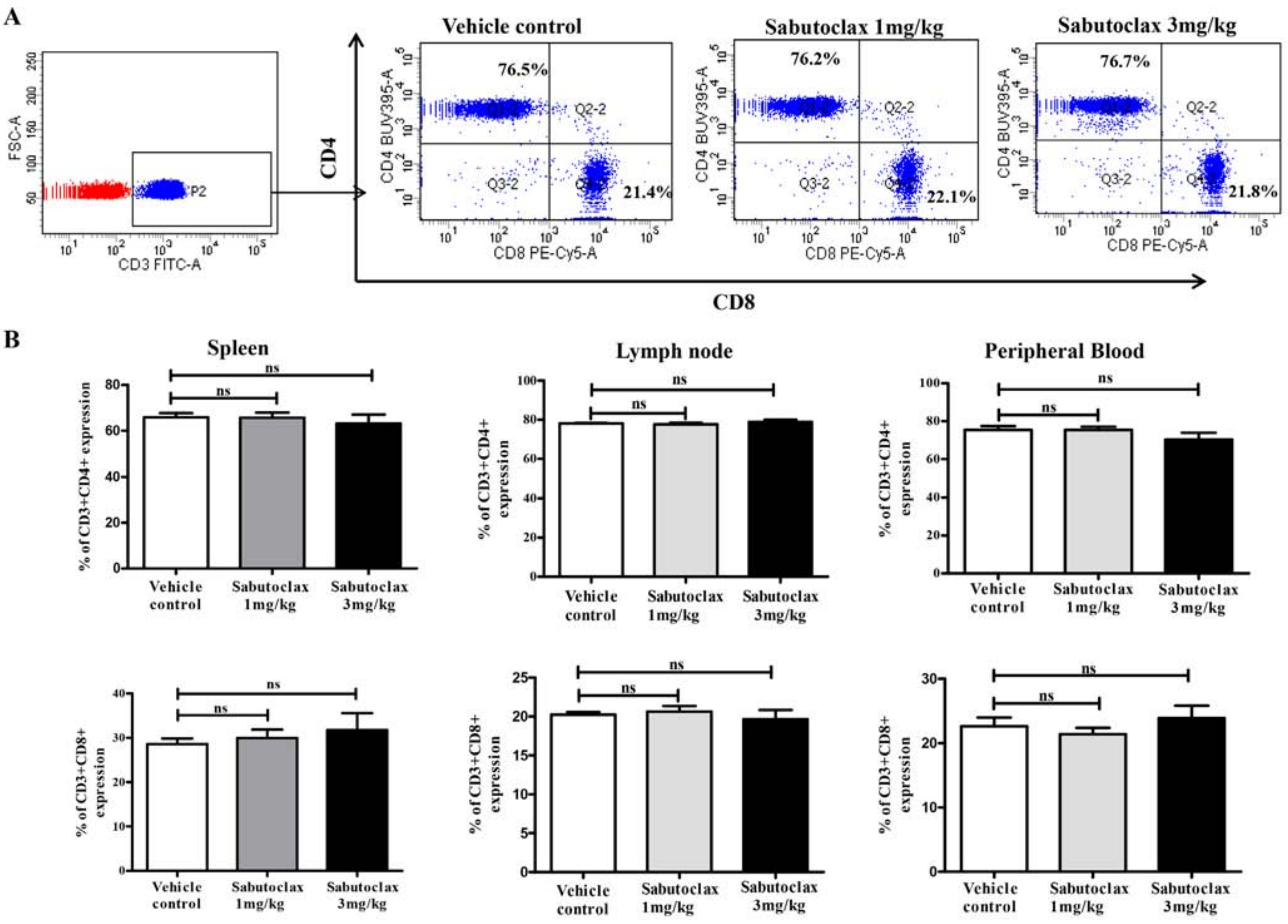

Supplementary Figure S6: Expression of CD4+ and CD8+ T lymphocytes in Sabutoclax treated BALB/c mice. BALB/c mice were injected IP with indicated concentration of Sabutoclax twice in week for 6 weeks. At the end of the experiment, expression of $CD3^+CD4^+$ and $CD3^+CD8^+$ T lymphocytes in peripheral blood, spleen and lymph nodes were analyzed by Flow cytometer as described in materials and methods. **A.** The gating strategy of CD3+ cell population (left) and one representative flow cytometry analysis of $CD3^+CD4^+$ or $CD3^+CD8^+$ cell population from one group (right) are indicated. **B.** Bar graphs represent the % of $CD3^+CD4^+$and $CD3^+CD8^+$ T lymphocytes in spleen, lymph node and peripheral blood. Error bars represent SE where $n = 5$ per mice. Statistical analysis were performed using Graph pad software (one way ANOVA) where $*p < 0.05$ considered as statistical significance. ns- Non significance.
